# Osteoinductivity and Antibacterial Properties of Strontium Ranelate-Loaded Poly(Lactic-co-Glycolic Acid) Microspheres With Assembled Silver and Hydroxyapatite Nanoparticles

**DOI:** 10.3389/fphar.2018.00368

**Published:** 2018-04-18

**Authors:** Zhenyang Mao, Yang Li, Yunqi Yang, Zhiwei Fang, Xuan Chen, Yugang Wang, Jian Kang, Xinhua Qu, Weien Yuan, Kerong Dai, Bing Yue

**Affiliations:** ^1^Shanghai Key Laboratory of Orthopedic Implants, Department of Orthopedic Surgery, Shanghai Ninth People’s Hospital, Shanghai Jiao Tong University School of Medicine, Shanghai, China; ^2^Department of Orthopaedic Surgery, Renji Hospital, Shanghai Jiao Tong University School of Medicine, Shanghai, China; ^3^Department of Orthopaedic Surgery, Shanghai Xinhua Hospital, Shanghai Jiao Tong University School of Medicine, Shanghai, China; ^4^School of Pharmacy, Shanghai Jiao Tong University, Shanghai, China

**Keywords:** PLGA, hydroxyapatite, silver, nanoparticles, strontium ranelate, MC3T3-E1 cell, osteogenesis, antibiosis

## Abstract

Bone-related infection rates are 4–64% in long open bone fractures and nearly 1% in joint-related surgeries. Treating bone infections and infection-related bone loss is very important. The present study prepared strontium ranelate (SR)-loaded poly(lactic-co-glycolic acid) (PLGA) microspheres (PM) with assembled silver nanoparticles (AgNPs) and hydroxyapatite nanoparticles (HANPs) (SR-PM-Ag-HA) through a novel solid-in-oil nanosuspension (S/O/N) method to achieve osteoinductivity and antibacterial properties. We evaluated the microstructure, drug release, biocompatibility, osteoinductivity, and antibacterial activity *in vitro*. The microspheres showed a stable shape and size. The cumulative drug release reached a maximum of ∼90% after 22 days. All groups loaded with SR enhanced MC3T3-E1 cell proliferation to a greater degree than pure PM. The osteoinductivity behavior was investigated by ALP staining and real-time PCR of osteogenic differentiation marker genes. The antibacterial activity was evaluated using antibacterial ability and biofilm formation assays. SR-PM-Ag-HA greatly enhanced osteogenic differentiation and showed excellent antibacterial properties. These results indicated that SR-PM-Ag-HA could be biocompatible and suitable for drug delivery, osteoinduction, and antibiosis, and therefore, have potential applications in the treatment of bone-related infections and promotion of bone formation at infected sites.

## Introduction

Bone-related infection rates are 4–64% in long open bone fractures and nearly 1% in joint-related surgeries. The best strategy of treating bone-related infections remains controversial. The application of systemic antibiotics has various complications and frequently, the local effective bactericidal concentration is unsuitable. Antibiotic-loaded bone cement bead chains are commonly applied as a local treatment. However, these materials cannot be biodegraded or absorbed by the body and need to be removed in a second operation. Meanwhile, the local burst release of antibiotics and their inability to treat drug-resistant bacteria restrict their clinical application. Moreover, bone absorption and bone loss are usually associated with bone-related infections. Currently, systemic or local antibiotic therapy does not improve osteogenesis. The treatment of both the bone infection and infection-related bone loss is equally important. Therefore, biodegradable biological materials loaded with antibiotics and active osteogenic elements, such as calcium phosphate cement, poly(lactic acid), poly(glycolic acid), polymer poly(lactic-co-glycolic acid) (PLGA), and various other polymers and nanoparticles, has proliferated in recent years.

PLGA is a promising material for clinical applications and has been used as a carrier for the delivery of proteins, drugs, and many other macromolecules, and as a scaffold for tissue engineering (Jain, 2000; Bouissou et al., 2006; Chan et al., 2009; Loo et al., 2010; Wang et al., 2010; Zhao et al., 2013; Zhou et al., 2013). PLGA has a high biocompatibility and hence, is a popular material for researching bone repair. However, PLGA has low hydrophilicity compared to other biodegradable biomaterials, which can negatively impact drug release, and cell attachment, proliferation, and differentiation. Moreover, most of the degradation products of PLGA are acidic, which may potentially cause inflammatory responses and reduce osteoinductivity (Arnett, 2008; Zhai et al., 2014; Liu et al., 2015).

Hydroxyapatite has been widely used in reconstructive bone surgery due to its good osteoinductive properties. Nano-sized HA, such as HANPs has notable physiochemical properties, such as a flexible structure, insensitivity to dissolution/growth, unique mechanical properties, and promotion of cell differentiation and proliferation. When PLGA is used as a drug delivery carrier, HANPs can ameliorate the hydrophilic environment, theoretically promoting drug release, neutralizing the acidity of PLGA degradation products, and improving the activity of osteoclasts.

In recent years, silver nanoparticles (AgNPs) have been widely developed and characterized at the nanoscale level, and have shown excellent antibacterial activity (Rai et al., 2009; Duran et al., 2016). Various studies reported AgNPs with good biocompatibility and antibacterial properties (Zheng et al., 2012; Tilmaciu et al., 2015; Cheng et al., 2016), with broad spectrum behavior against Gram-positive and Gram-negative bacteria, including antibiotic-resistant strains. In this study, we chose AgNPs as an antimicrobial agent for the treatment of mixed and drug-resistant bacterial infections, as it shows good antimicrobial activity and has been approved in medicine for many years (Lansdown, 2002; Castellano et al., 2007; Hiro et al., 2012). SR is a medication for osteoporosis, which both reduces bone resorption and simultaneously increases new bone deposition by osteoblasts, i.e., a dual action bone agent.

In the present study, we aimed to combine all the advantages of the materials discussed above. Hence, we fabricated SR-loaded PLGA microspheres assembled with AgNPs and HANPs (SR-PM-Ag-HA) to achieve osteoinductivity and antibacterial properties. We compared this composite material with PM, SR-PM, and SR-PM-Ag samples and evaluated their microstructures, SR release, biocompatibility, osteoinductivity, and *in vitro* antibacterial activity.

## Materials and Methods

**FIGURE 1:**
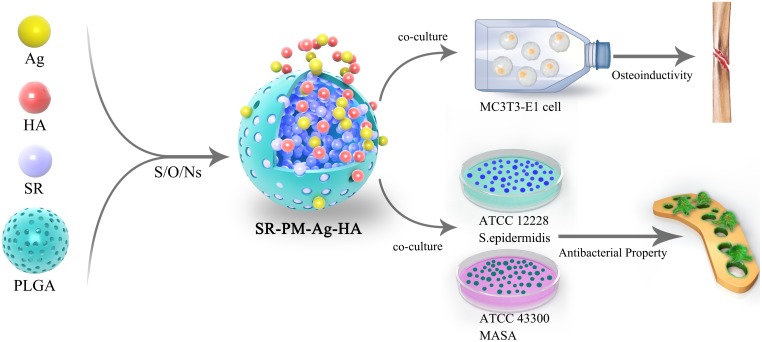
Schematic diagram of the overall experimental protocol.

The overall experiment protocol is shown schematically in **Figure 1**.

### Preparation of Microspheres

#### Materials

PLGA (65:35; molecular weight, Mw 20 kDa) was obtained from Lakeshore Biomaterials (Germany). PVA (87–89% hydrolyzed, Mw 31000–50000), HANPs, SR, AgNO_3_, and NaBH_4_ were supplied by Sigma (United States). Dichloromethane and other reagents used were all of analytical grade.

#### Preparation of PVA + HA + Ag Nanosuspension

We prepared the PVA + HA + Ag nanosuspension by adding 50 mg AgNO_3_ to 4.5 ml of an aqueous solution of 2% (w/w) PVA under vigorous stirring using a Fluko FA25 homogenizer at 2000 rpm. Ag nanosuspensions with different colors were then obtained by dropping various amounts of reducing agent (NaBH_4_) into AgNO_3_ solutions. We chose the yellow nanosuspension due to its small Ag nanoparticle size. After adding 100 mg HANPs, the PVA + HA + Ag nanosuspension was prepared.

#### Preparation of PLGA Microspheres

We applied the double emulsion in water-in-oil-in-water (W/O/W) method to fabricate the PM. We added 1 ml of water which had been deionized to 4 ml of 6.25% (w/w) PLGA methylene chloride solution. The first W/O emulsion was prepared with a Powergen 700 homogenizer (Fisher Scientific Co., United States) for 3 min at 5000 rpm. Then, the emulsion was immediately poured into a beaker with 200 ml of 1% (w/w) PVA solution and subsequently re-emulsified at 200 rpm for 4 h using an overhead propeller (LR-400A, Fisher Scientific Co., United States). The microspheres were separated by centrifugation after evaporation of the solvent. After washing three times in distilled water and freeze drying, PM were achieved.

#### Preparation of SR-Loaded PLGA Microspheres

A solution containing 250 mg of PLGA, 13.4 mg of freeze-dried SR powder, 1 ml of deionized water containing NH_4_HCO_3_ (10%, w/v), and 4 ml of methylene chloride was prepared. The primary S/O emulsion was prepared through the same previous process. The microspheres were separated by centrifugation after evaporation of the solvent. The microspheres were then washed three times with distilled water and then freeze dried. Next, 5% SR-loaded PLGA microspheres (SR-PM) were prepared.

#### Preparation of Microspheres by S/O/N

We added 10% (w/w) PLGA solution dissolved in dichloromethane to the previously prepared nanosuspension. Then, this O/N emulsion was transferred to 1000 ml (0–4°C) of 5% (w/w) sodium chloride solution and aged in an electromotive stirrer (Xinhang JJ-1, Jintan Xinhang Co. Ltd., China) under gentle stirring (100 rpm) for 2 h in order to evaporate the solvent to harden the mixture. Then the hardened microspheres were rinsed with distilled water, which were then freeze dried before storage.

#### Preparation of SR-Loaded PLGA Microspheres With AgNPs (and HANPs) by S/O/N

We added, 13.4 mg of SR, and 1 ml of deionized water containing NH_4_HCO_3_ (10%, w/w) to 4 ml of 6.25% (w/w) PLGA methylene chloride solution. The primary S/O emulsion was produced using the Powergen 700 homogenizer at 5000 rpm for 3 min. Then, the emulsion was immediately poured into a beaker with 200 ml of the prepared 2% (w/w) PVA and Ag nanosuspension (S/O/N), then re-emulsified at 200 rpm for 4 h with the LR-400A overhead propeller. The microspheres were separated by centrifugation after evaporation of the solvent. The microspheres were then washed three times with distilled water and freeze dried to prepare 5% SR-loaded PLGA microspheres with assembled Ag (1%) nanoparticles (SR-PM-Ag). In the same way, we prepared 5% SR-loaded PLGA microspheres assembled with Ag (1%) and HA (2%) nanoparticles (SR-PM-Ag-HA).

### Characterization

#### Particle Size and Morphology Characterization

The particle sizes of the HANPs and AgNPs used in this study were measured using a particle-size analyzer (Brookhaven Instruments). In addition, an FEI Sirion 200 scanning electron microscope (SEM; United States) was used to obtain images of HANP, AgNP, PM, SR-PM, SR-PM-Ag, and SR-PM-Ag-HA samples. All specimens were coated by gold evaporation at 5 keV under an argon atmosphere before SEM tests.

#### Encapsulation Efficiency Test

A 15 mg sample of drug-loaded PLGA microspheres (SR-PM, SR-PM-Ag, or SR-PM-Ag-HA) was immersed in 5 ml dichloromethane and then centrifuged for 5 min at 12000 rpm to remove the PLGA. Next, it was re-dissolved in 3 ml phosphate-buffered saline at pH 7.4, and then centrifuged for 5 min at 12000 rpm. A supernatant liquid was prepared for HPLC tests to confirm the SR content. The efficiency of SR encapsulation in the microspheres was calculated as follows:

Encapsulationefficiency(%) = P/Pt × 100

where Pt is the total theoretical weight of SR and P is the total actual weight of SR encapsulated into the PLGA microspheres. The encapsulation efficiency was calculated from the average of three experiments and its standard deviation for drug loading was determined.

In order to analyze the formulated SR, we developed a room-temperature reverse-phase (RP)-HPLC method capable of detecting UV. A Diamonsil C18 column (250 mm × 4.6 mm, 5 μm) was used as the stationary phase, while the mobile phase was a 0.2% acetic acid buffer (adjusted to pH = 5.5 using triethylamine) in methanol (95:5). The eluent was placed in the monitor of a UV detector with a flow rate of 1 at 234 nm. A linear response was observed using this method, which exceeded the concentration range of 10–200 μg/ml. The detection and quantification limits were 20 and 50 ng, respectively.

#### Elemental Concentrations

The concentrations of key elements (Sr for SR-PM, SR-PM-Ag, and SR-PM-Ag-HA; P and Ca for SR-PM-Ag-HA; and Ag for SR-PM-Ag and SR-PM-Ag-HA) were investigated using polarized Zeeman AAS (Z-2000, Hitachi). We used these values to subsequently calculate the contents of SR, Ag, and HA in the microspheres.

#### pH Changes With PLGA Degradation

We incubated 150 mg of PLGA microspheres at 37°C in vials with 5 ml of phosphate-buffered saline (100 mM, pH 7.4) which was shaken constantly. Next, 50 μL of penicillin (100 U/ml) and streptomycin (100 g/ml) were added to the buffer to avoid microorganism growth. The pH of the buffer was determined using an FE20 digital pH meter (Mettler Toledo) every 5 days. This experiment was repeated three times to acquire average pH profiles.

#### *In Vitro* Drug Release

The drug-loaded PLGA microspheres (50 mg) including SR-PM, SR-PM-Ag, and SR-PM-Ag-HA were incubated in the same way as described in Section “pH Changes With PLGA Degradation.” To investigate the release of SR, a fresh buffer was used to replace the release medium on scheduled dates and the contents of SR were assayed with RP-HPLC (the same method as used for the encapsulation efficiency tests). Each measurement was repeated in triplicate for every sample to determine average release profiles.

### Proliferation and Osteogenic Differentiation of MC3T3-E1 Cells

#### MC3T3-E1 Cell Proliferation

To culture the MC3T3-E1 cells, an α-minimal medium (α-MEM) was used (Gibco, United States), where 100 mg/ml streptomycin, 100 U/ml penicillin, and 10% fetal bovine serum (FBS) were supplemented (all from Hyclone, United States). In all of the experiments, culturing was undertaken at 37°C with 5% CO_2_ in a humid atmosphere, where the medium was changed every 3 days.

The effects of PM, SR-PM, SR-PM-Ag, and SR-PM-Ag-HA on cell proliferation were determined using a CCK-8 assay following the instructions of the manufacturer. MC3T3-E1 cells were plated at a density of 3 × 10^3^ cells in 96-well plates with each well tested in triplicate. The cells were treated using four materials with a concentration of 5 mg/ml 24 h later. Next, 10 μL CCK-8 was added to the wells and the plates were incubated for another 2 h at 37°C. Then, the optical density (OD) of the samples was measured using an ELX800 absorbance microplate reader (Bio-Tek Instruments, United States) at a wavelength of 450 nm (calibrated at 650 nm).

#### Osteogenic Differentiation of MC3T3-E1 Cells

The MC3T3-E1 cells were co-cultured with 5 mg/ml of either PM, SR-PM, SR-PM-Ag, or SR-PM-Ag-HA in a 12-well plate at a density of 5 × 10^5^ cell/ml. The medium was changed every 3 days. After culturing of 3 days in the basic medium, the cells in the osteogenic medium were subjected to osteogenic differentiation. The osteogenic medium consisted of a basic culture medium supplemented with 50 mM L-ascorbic acid, 10^-8^ M dexamethasone, and 10 mM β-glycerophosphate (β-GP) (all from Sigma, United States). At each sampling time, the cells were separated with trypsin from the disk samples, and transferred to a 24-well plate.

Cell staining with ALP was performed following the instructions of the manufacturer (Rainbow, China). A RT-PCR was used to analyze the expression of osteogenic differentiation marker genes, such as ALP, RunX-2, OCN, and pre-procollagen type I (Col I). An extraction method for TRIzol (Invitrogen) was used to isolate total RNA, and the synthesis of cDNA was performed in accordance with the provided protocol (OmniScript Reverse Transcription Kit; Qia-gen, United States). SybrGreen (BioRad, United States) was used to quantify the starting mRNA using a standard curve method provided with the Perkin-Elmer Gene AMP PCR system 2400 (Kasten et al., 2003).

### Bacterial Culture and Antibacterial Effect Assay

#### Preparation of Bacteria

*Staphylococcus epidermidis* (ATCC12228) and methicillin-resistant *Staphylococcus aureus*, (MRSA; ATCC43300) were obtained from the American Type Culture Collection (ATCC). Bacteria were cultured in a tryptone soy broth (TSB) while shaking at 120 rpm at 37°C for 12–16 h. The concentration of bacteria was measured using the OD method following McFarland and diluted to 1 × 10^6^ colony-forming units (CFU)/mL for testing.

#### Antibacterial Activity

The antibacterial activities of the four kinds of microspheres against each bacteria strain were determined using spread plate analysis (van de Belt et al., 2001; Dunne et al., 2007). The concentrations of strains ATCC12228 and ATCC43300 were all diluted to a density of 1 × 10^6^ CFU/mL in TSB. PM, SR-PM, SR-PM-Ag, and SR-PM-Ag-HA were added to the bacterial suspensions with concentrations of 5 mg/mL. After being shaken at 120 rpm at 37°C for 24 h, the solutions were serially diluted 10-fold, and 500 μL of each suspension was plated onto tryptone soy agar (TSA) and incubated at 37°C for 24 h. This step was repeated three times. The CFUs on the TSA were then counted following incubation. The OD values were measured using an absorbance microplate reader at 570 nm.

#### Determination of Biofilm Formation

We placed 5 mg PM, SR-PM, SR-PM-Ag, and SR-PM-Ag-HA in a new 24-well plate containing one Ti disk (φ = 15 mm) per well. One milliliter of TSB bacterial suspension (1 × 10^6^ CFU/mL) was added. Then, the 24-well plate was incubated at 37°C for 24 h. After gentle washing three times with PBS, the Ti disks were stained with 300 μL combination dye (LIVE/DEAD Baclight Bacteria Viability kit, Molecular Probes, L13152). Subsequently, the stained disks were viewed by confocal laser scanning microscopy (CLSM; Leica TCS SP2; Leica Microsystems, Germany). Fluorescent green represented the viable bacteria with intact cell membranes, while fluorescent red represented for non-viable bacteria with broken membranes.

Bacterial biofilms were stained using the crystal violet method. We placed 5 mg of PM, SR-PM, SR-PM-Ag, and SR-PM-Ag-HA into a new 24-well plate containing one Ti disk (φ = 15 mm) per well. Bacterial suspensions with a concentration of 1 × 10^4^ CFU/mL were added to the plates (0.5 ml/well) and incubated at 37°C for 2, 5, and 8 h. The disks were moved into fresh wells after the medium was removed, followed by washing six times with PBS. Then, the disks were stained in a new well with 1 ml PBS containing 100 μL of 1% crystal violet (Sigma-Aldrich, United States). After shaking at room temperature for 15 min, the disks were washed three times with PBS and crystal violet, and solubilized by adding 2 mL 95% ethanol with rocking for 15 min. OD measurements were undertaken as previously described.

### Statistical Analysis

Statistical analyses were performed using SPSS 19.0 software. Continuous variables were expressed as a mean ± standard deviation (SD). Between-group differences for demographic and clinical variables were evaluated using one-way analysis of variance (ANOVA), with least squared difference (LSA) *post hoc* test used for variables with homogeneity of variances or Dunnett’s test for variables with heterogeneity of variances. A value of *P <* 0.05 was considered statistically significant.

## Results

### Characterization

#### Particle Morphology

**FIGURE 2:**
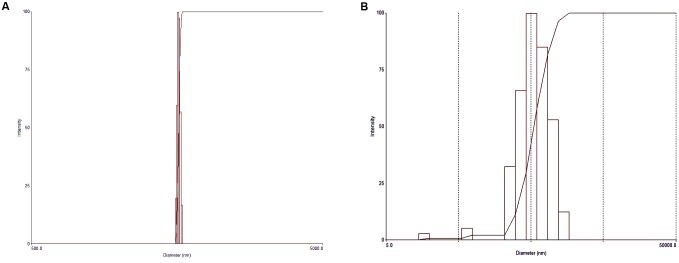
Particle sizes of **(A)** HANPs and **(B)** AgNPs.

**FIGURE 3:**
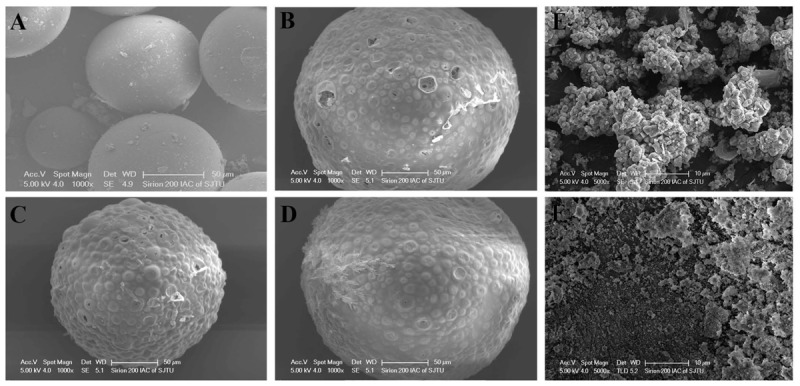
SEM images of **(A)** PM, **(B)** SR-PM, **(C)** SR-PM-Ag, **(D)** SR-PM-Ag-HA, **(E)** HANPs, and **(F)** AgNPs.

Particle size measurements of the HANPs indicated an average diameter of 1986.8 nm, with a polydispersity of 0.005, while the AgNPs had an effective diameter of 469.3 nm and polydispersity of 0.249 (**Figure 2**). The morphologies of HANPs, AgNPs, PM, SR-PM, SR-PM-Ag, and SR-PM-Ag-HA were characterized using SEM. As is shown in **Figure 3**, the NPs were assembled on the surface of the microspheres. The PM samples showed good surfaces while SR-PM-Ag and SR-PM-Ag-HA showed rough surfaces.

#### Encapsulation Efficiency

According to the equation defined in the methods section. The encapsulation efficiencies for SR-PM, SR-PM-Ag, and SR-PM-Ag-HA were 54.02 ± 9.26%, 56.25 ± 9.93%, and 55.78 ± 9.87%, respectively.

#### Microsphere Composition

**FIGURE 4:**
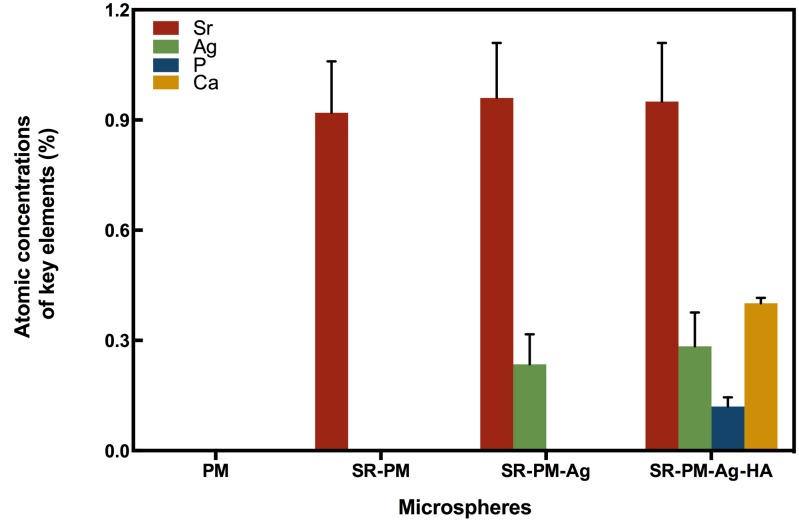
Atomic absorption spectroscopy composition of the four types of microspheres, where the data are represented as mean ± SD.

The compositions of the four kinds of microspheres analyzed using AAS are shown in **Figure 4**. The PM composition did not contain Sr, Ag, P, or Ca, while the composition of SR-PM contained Sr (0.92 ± 0.14%) but not Ag, P, or Ca. The composition of SR-PM-Ag contained Sr (0.96 ± 0.15%), Ag (0.235 ± 0.082%), but not P or Ca. The composition of SR-PM-Ag-HA contained Sr (0.95 ± 0.16%), Ag (0.248 ± 0.092%), P (0.12 ± 0.025%), and Ca (0.401 ± 0.015%). These results confirmed that SR was successfully encapsulated into the microspheres and Ag and HA was present on the surface of the microspheres.

#### pH Changes With PLGA Degradation

**FIGURE 5:**
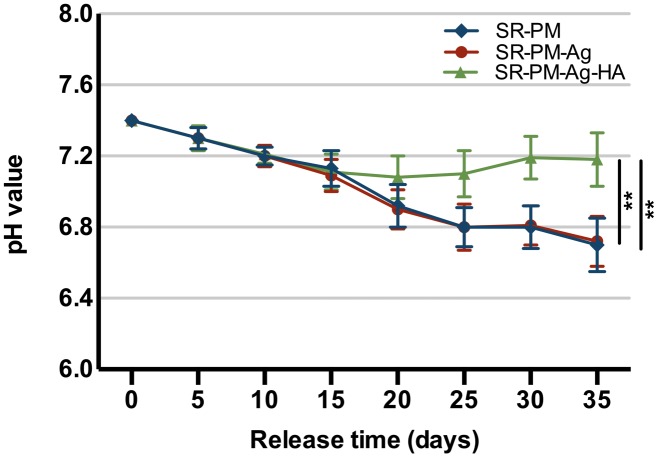
pH changes with PLGA degradation. The data are represented as mean ± SD. (^∗∗^ for *P* < 0.01).

In a water environment, PLGA biodegrades by hydrolysis of its ester linkages into lactic acids and glycolic acids. The microspheres with HANPs produced in this study have the advantage of being able to neutralize the acidity of PLGA degradation products. In **Figure 5**, it can be seen that after 2 weeks of degradation, the pH values significantly decreased, and there was no significant difference between the two formulations (with and without HANPs) (*P* > 0.05). Over the next 3 weeks, the microspheres with HANPs stabilized the pH above 7.1, which was significantly higher than the values for the SR-PM or SR-PM-Ag samples (*P* < 0.05). It is hypothesized that the HANPs were released from the microspheres and reacted with hydrogen ions dissociated from the lactic and glycolic acids as HANPs is alkaline.

#### *In Vitro* Drug Release

**FIGURE 6:**
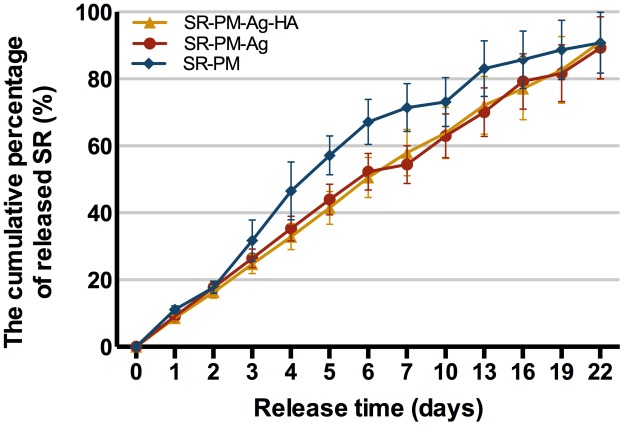
Cumulative drug release (%). The data are represented as mean ± SD.

Strontium ranelate release curves are shown in **Figure 6** gently increasing curves were observed. The percentage cumulative release values for SR-PM, SR-PM-Ag, and SR-PM-Ag-HA were all a maximum of ∼90% of the total loaded drug after 22 days. All three SR-loaded microsphere samples released SR without a drug release burst, while those with NPs assembled on the surface showed the desired zero-order release.

### Proliferation and Osteogenic Differentiation of MC3T3-E1 Cells

#### MC3T3-E1 Cell Proliferation

**FIGURE 7:**
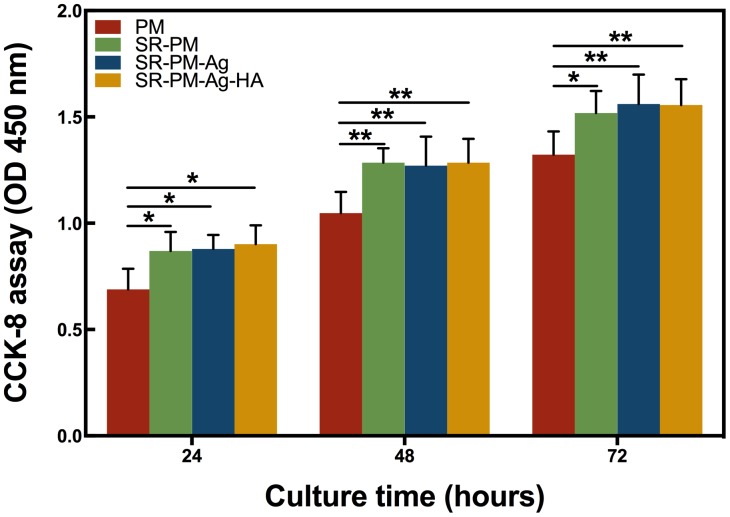
CCK-8 assay results of the MC3T3-E1 cells co-cultured with PM, SR-PM, SR-PM-Ag, and SR-PM-Ag-HA at each time. The data are represented as mean ± SD. (^∗^ for *P* < 0.05, ^∗∗^ for *P* < 0.01).

The CCK-8 assay results for the MC3T3-E1 cells co-cultured with PM, SR-PM, SR-PM-Ag, and SR-PM-Ag-HA for different periods are shown in **Figure 7**. The MC3T3-E1 cells proliferated sufficiently. None of the microsphere samples exhibited significant cytotoxicity for MC3T3-E1 cells. After co-culturing for 3 days, SR-PM, SR-PM-Ag, and SR-PM-Ag-HA showed a greater potential to stimulate MC3T3-E1 cell proliferation compared to PM (*P* < 0.05), while no significant difference was observed between SR-PM, SR-PM-Ag, and SR-PM-Ag-HA samples (*P* > 0.05). These results showed that the biocompatibility of all microspheres was acceptable.

#### Osteogenic Differentiation of MC3T3-E1 Cells

**FIGURE 8:**
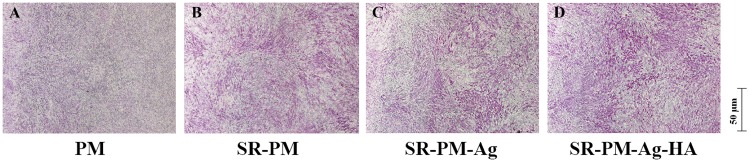
ALP staining results of the MC3T3-E1 cells after co-culturing with **(A)** PM, **(B)** SR-PM, **(C)** SR-PM-Ag, and **(D)** SR-PM-Ag-HA for 7 days in the osteogenic medium.

The osteoinductivity behavior of PM, SR-PM, SR-PM-Ag, and SR-PM-Ag-HA was evaluated using osteogenic differentiation of MC3T3-E1 cells. **Figure 8** shows typical ALP staining results of MC3T3-E1 cells after co-culturing for 7 days in the osteogenic medium. The ALP staining intensity of the MC3T3-E1 cells with SR-PM, SR-PM-Ag, and SR-PM-Ag-HA was stronger than that for PM. The ALP staining intensity of the MC3T3-E1 cells with SR-PM-Ag-HA was stronger than with SR-PM and SR-PM-Ag, while SR-PM and SR-PM-Ag could not be distinguished. These results demonstrated that SR-PM-Ag and SR-PM promote osteogenic differentiation in the MC3T3-E1 cells to a greater degree than PM, which was further enhanced for SR-PM-Ag-HA.

**FIGURE 9:**
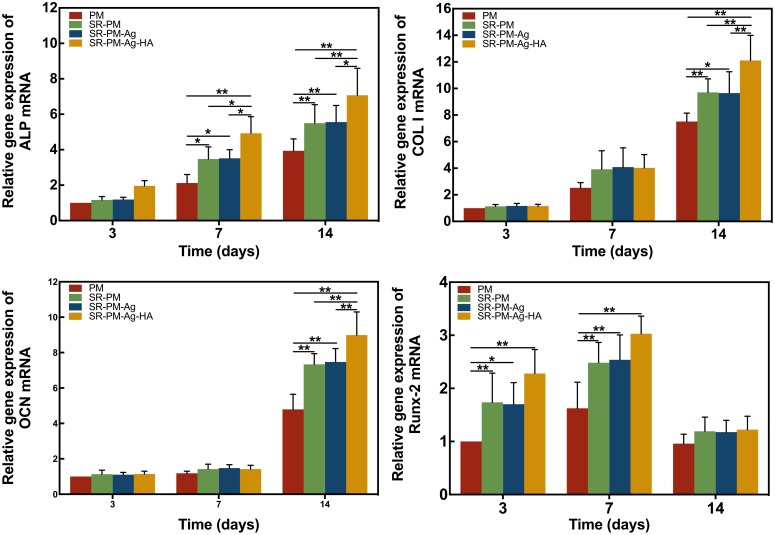
Relative expression of osteogenic differentiation marker genes. The data are represented as mean ± SD. (^∗^ for *P* < 0.05, ^∗∗^ for *P* < 0.01).

The osteogenic differentiation marker genes ALP, Col I, OCN, and RunX-2 are used as markers for osteoblastic activity (Ducy et al., 1997; Franceschi, 1999; Wang et al., 2003). After 3 days, the expression of ALP had no significant difference among the MC3T3-E1 cells co-cultured with the four different microspheres (*P* > 0.05). After 7 and 14 days, the expression of ALP of the MC3T3-E1 cells co-cultured with SR-PM-Ag-HA was consistently higher than the other three kinds of microspheres (*P* < 0.05). Higher levels of Col and OCN were observed for SR-PM and SR-PM-Ag at 14 days compared to PM (*P* < 0.05), while those for SR-PM-Ag-HA were the highest (*P* < 0.05). The highest level of RunX-2 was observed for SR-PM-Ag-HA (*P* < 0.05). After 14 days, the levels of Runx-2 decreased. No significant differences in marker genes expression were observed between SR-PM and SR-PM-Ag (*P* > 0.05), as shown in **Figure 9**.

### Antibacterial Properties

**FIGURE 10:**
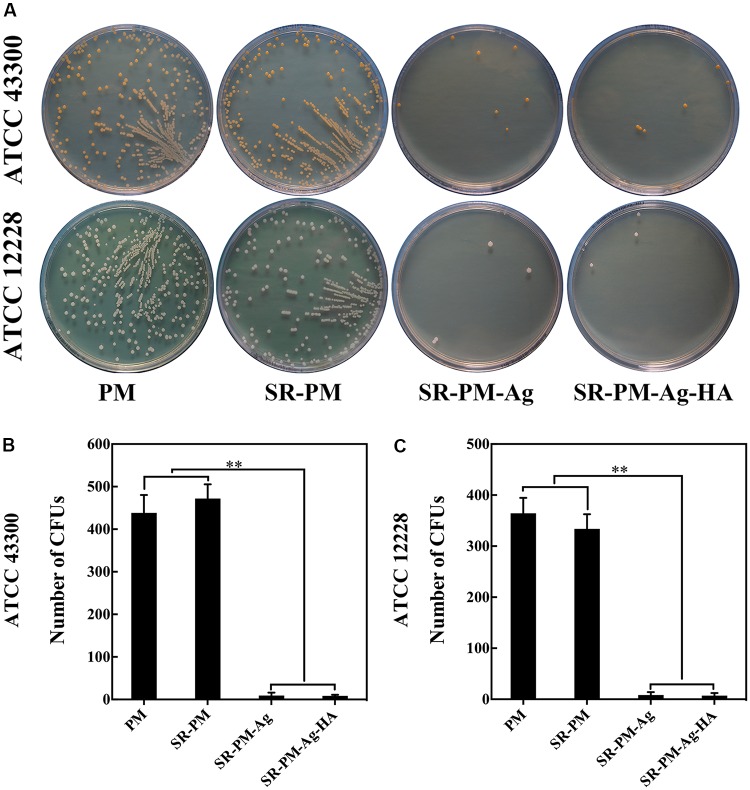
**(A)** Surviving bacteria CFU on the TSA after co-culturing after 24 h. Colonies of viable **(B)** ATCC43300 and **(C)** ATCC12228 bacteria on different TSA samples. The data are represented as mean ± SD. (^∗∗^ for *P* < 0.01).

The antibacterial activities of the four kinds of microspheres against each bacterial strain. The surviving bacteria CFUs were counted on the TSA after culturing for 24 h. Fewer CFUs were observed for SR-PM-Ag and SR-PM-Ag-HA than for PM and SR-PM, as shown in **Figure 10** (*P* < 0.01); hence SR-PM-Ag and SR-PM-Ag-HA showed the best antibacterial properties.

**FIGURE 11:**
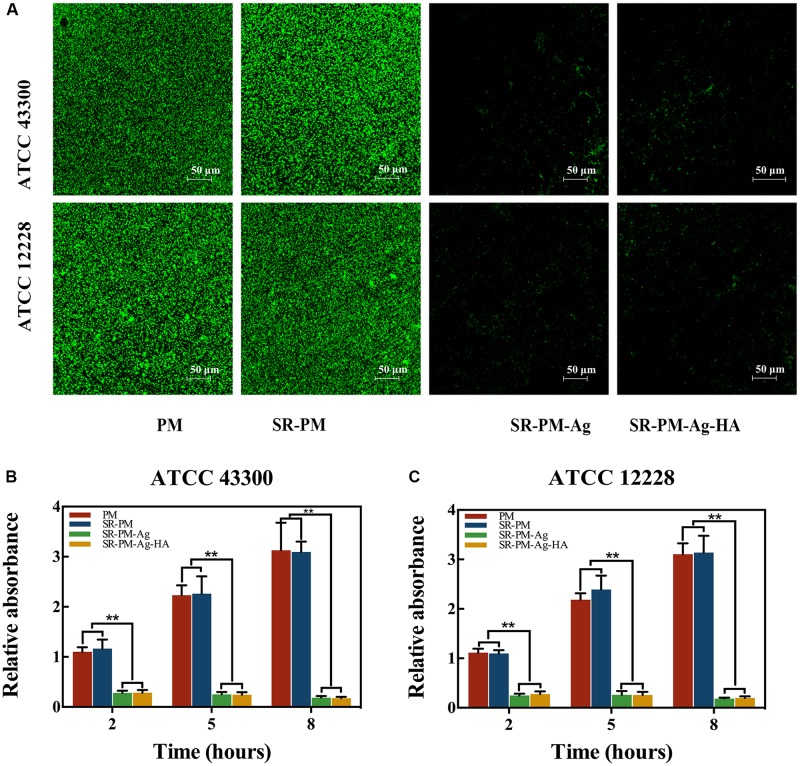
**(A)** CLSM results of biofilm formation of two bacteria strains on Ti disks after co-culturing with four different microspheres. Absorption of crystal violet staining intensity indicated biofilm formation by **(B)** ATCC43300 and **(C)** ATCC12228 after 2, 5, and 8 h of co-culturing. The data are represented as M ± SD. (^∗∗^ for *P* < 0.01).

Biofilm formation of two strains on Ti disks after co-culturing with the four kinds of microspheres is shown in **Figure 11A**, where viable cells were stained fluorescent green. It can be seen that fewer living bacteria colonies of either strain were visible after co-culturing with SR-PM-Ag or SR-PM-Ag-HA compared to co-culturing with PM or SR-PM. Intense fluorescence indicates a well-coated biofilm while weak fluorescence indicates little biofilm formation. The crystal violet staining intensity on the Ti disks surfaces with PM and SR-PM was much higher after 5–8 h than at 2–5 h (*P* < 0.01) (**Figures 11B,C**). The biofilm formation on Ti disks surfaces with SR-PM-Ag and SR-PM-Ag-HA was significantly reduced (*P* < 0.01), indicating that these materials greatly inhibited bacterial biofilm formation.

## Discussion

We successfully fabricated SR-loaded microspheres with and without HANPs or AgNPs assembled on the surface. We investigated the effects of PM, SR-PM, SR-PM-Ag, and SR-PM-Ag-HA on the biocompatibility, drug delivery, osteoinductivity, and antibacterial properties. We found that SR-loaded microspheres steadily released SR without a drug release burst. No significant cytotoxicity was observed for any samples. SR-PM-Ag-HA effectively promoted osteogenesis of MC3T3-E1 cells, as well and preventing bacterial adhesion and biofilm formation.

To date, microspheres have been developed by many different methods; the emulsion method is the most widely employed. Microspheres have been developed using many novel techniques (Cai et al., 2013; Hong et al., 2013; Jiang et al., 2016; Park et al., 2016; Wei et al., 2016; Xiao et al., 2016; Zhang et al., 2016). However, these methods failed to improve the hydrophobic properties of the microsphere surfaces, resulting in poor biocompatibility with biological tissue; the acidity of PLGA degradation products resulted in acid-sensitive drugs losing efficacy, inflammatory reaction, and problems with microspheres-high efficacy-loaded drug. Here, we presented a novel S/O/N method for preparing microspheres to address these issues. PVA, HA, and/or Ag nanosuspensions were used as surfactants; HA and Ag NPs were easily assembled on the microsphere surfaces due to the action of the surfactants. Moreover, the S/O/N method may have reduced drug on the microsphere surface, significantly reducing the burst release.

Hydroxyapatite nanomaterials incorporated with other materials have been reported (Wan et al., 2015; Ma et al., 2016; Wijesinghe et al., 2016). Hu et al. (2014) fabricated an injectable scaffold consisting of a HA-PLGA microsphere composite prepared by ionic bonding. They found that these microspheres clearly promoted osteoblast attachment, proliferation, and ALP activity. In this study, we combined HA and PLGA to successfully prepare microspheres. Some studies regarding HA reported good biocompatibility (Chen et al., 2015; Shen et al., 2015), consistent with our results. Furthermore, our osteogenic differentiation studies showed that HANPs contributed to stronger ALP staining of MC3T3-E1 cells with SR-PM-Ag-HA than SR-PM-Ag after 7 days. In addition, the MC3T3-E1 cells co-cultured with SR-PM-Ag-HA expressed all detected marker genes more efficiently than SR-PM-Ag. These findings indicated that SR released from SR-PM-Ag-HA, along with the HANPs, jointly stimulated *in vitro* osteoinductivity.

Several pharmacologic options are available for bone loss therapy, including the selective estrogen-receptor modulator, bisphosphonates, SR, calcitonin, and parathyroid hormone (PTH). Among these drugs, SR and PTH can promote osteogenesis, while the others only inhibit bone resorption. SR is a dual action bone agent and is unusual in that it both increases osteogenesis by osteoblasts and reduces bone resorption by osteoclasts (Atkins et al., 2009; Brennan et al., 2009; Hurtel-Lemaire et al., 2009). Here, CCK-8 assays indicated that SR-PM, SR-PM-Ag, and SR-PM-Ag-HA showed no cytotoxicity and could all significantly stimulate proliferation of MC3T3-E1 cells compared to PM. Caverzasio (2008) and Takaoka et al. (2010) showed that SR had a positive effect on proliferation of pre-osteoblasts, consistent with our results. Chattopadhyay et al. (2007) reported that SR affects cellular processes via interactions between the sensing receptor and calcium, leading to enhanced gene expression of erg1 and c-fos, both involved in osteoblast proliferation regulation.

Alkaline phosphatase is a marker gene which is expressed mainly in matrix vesicles or on cell surfaces. In the process of osteogenic differentiation, the expression of ALP increases slowly to a maximum when the cells reach matrix maturation, and subsequently decreases at the matrix mineralization stage. The ALP staining intensity of the MC3T3-E1 cells with SR-PM or SR-PM-Ag was stronger than with PM, which meant that released SR could notably facilitate osteogenesis of MC3T3-E1 cells *in vitro*. Col I is a protein that is the most abundant component in mature osteoblasts. OCN is a late bone marker and plays an important role in regulating the growth and formation of bone minerals, indicating the final stage of osteogenesis. These three marker genes may be expressed continually during osteogenesis. As a fundamental factor for the formation of bone and hypertrophic cartilage, Runx-2 is expressed early in the osteogenesis process (Ducy et al., 1997; Tu et al., 2016; Wu et al., 2016). Studies of the expression of these marker genes reflecting bone formation showed similar osteogenic differentiation of MC3T3-E1 cells co-cultured with SR-PM and SR-PM-Ag, indicating that AgNPs might not stimulate osteogenic differentiation of MC3T3-E1 cells. Our results indicated that SR stimulated osteoblast differentiation. Bonnelye et al. (2008) also reported that SR stimulates osteoblastic differentiation markers [such as ALP, bone sialoprotein (BSP), and OCN] in primary murine osteoblasts. Our results also agreed with others reporting that SR induces osteogenesis by increasing preosteoblast-osteoblast differentiation, bone matrix synthesis, and mineralization (Canalis et al., 1996; Ammann, 2006; Marie, 2006; Choudhary et al., 2007; Yang et al., 2011; Querido et al., 2015).

Many studies have demonstrated that AgNPs could be effective against broad-spectrum bacteria (Pal et al., 2007; Navarro et al., 2008; Manke et al., 2013; dos Santos et al., 2014; Rajeshkumar and Malarkodi, 2014; Wu et al., 2015), which should be ascribed to the effect of silver ions which disrupt the outer membrane of target cells. AgNPs were confirmed to interact with the building elements of the bacterial membrane, causing damage to the cell (Sondi and Salopek-Sondi, 2004). Panacek et al. (2006) found high antimicrobial and antibacterial activity of AgNPs against both Gram-positive and Gram-negative bacteria, where the antibacterial activity was found to be dependent on the NP size. Stevanovic et al. (2013) developed a PLGA-AgNP composite that showed superior and extended antibacterial activity against Gram-positive methicillin-resistant *Staphylococcus aureus* and Gram-negative *Escherichia coli*. Here, we used yellow AgNPs due to their small size. SR-PM-Ag and SR-PM-Ag-HA had better antibacterial efficiencies than PM and SR-PM. Bacterial adhesion followed by biofilm formation are the initial key steps in bacterial colonization resulting in bone infection. Once the biofilm generates, bacteria can adhere more easily and are difficult to eliminate (Donlan and Costerton, 2002). We observed that there were fewer surviving colonies on the TSA with SR-PM-Ag and SR-PM-Ag-HA than for PM and SR-PM. More viable cells were observed in CLSM images for Ti disks co-cultured with PM and SR-PM compared with those co-cultured with SR-PM-Ag and SR-PM-Ag-HA. The highly enhanced antibacterial properties of SR-PM-Ag and SR-PM-Ag-HA were ascribed to the AgNPs assembled on the surface of PLGA microspheres.

The limitations of our study should be acknowledged. First, the use of human bone marrow mesenchymal stem cell would have been better for *in vitro* experiments than MC3T3-E1 cells. In addition, the use of other sample groups, including a different kind of control sample such as PLGA microspheres with assembled HANPs loading SR would have further verified our results. Furthermore, future *in vivo* studies would be interesting to verify the results presented here. The specific mechanism of antibacterial activity of AgNPs would be interesting to explore in the future.

## Conclusion

We successfully fabricated SR-loaded PLGA microspheres assembled with HANPs and/or AgNPs. None of the microspheres used in this study showed cytotoxicity. SR-PM, SR-PM-Ag, and SR-PM-Ag-HA showed adequate drug release performance and could effectively promote the proliferation of MC3T3-E1 cells. SR-PM-Ag-HA significantly enhanced osteogenic differentiation of MC3T3-E1 cells and showed good antibacterial properties.

## Author Contributions

ZM, YY, WY, and KD designed the work. ZM, ZF, XC, YW, JK, BY, and XQ performed the experiments and analyzed the data. ZM, YL, and ZF were major contributors in writing the manuscript. All authors read and approved the final manuscript and contributed equally to this work.

## Conflict of Interest Statement

The authors declare that the research was conducted in the absence of any commercial or financial relationships that could be construed as a potential conflict of interest.

## Abbreviations

AAS: atomic absorption spectrophotometry
AgNPs: Ag nanoparticles
ALP: alkaline phosphatase
Col I: collagen type I
HA: hydroxyapatite
HANPs: HA nanoparticles
HPLC: high-performance liquid chromatography
OCN: osteocalcin
PM: PLGA microspheres
PVA: polyvinyl alcohol
RP-HPLC: reversed-phase high-performance liquid chromatography
RT-PCR: real-time polymerase chain reaction
Runx-2: runt-related transcription factor 2
S/O/N: solid-in-oil-in-nanosuspension
SR: strontium ranelate
SR-PM: SR-loaded PM
SR-PM-Ag: SR-PM with AgNPs assembled on the surface
SR-PM-Ag-HA: SR-PM with AgNPs and HANPs assembled on the surface.
